# Prognostic value of EIF5A2 in solid tumors: A meta-analysis and bioinformatics analysis

**DOI:** 10.1515/med-2024-0962

**Published:** 2024-05-16

**Authors:** Jianwen Fang, Tianze Yu, Xiaocong Jiang, Yuexin Lu, Xi Shang, Haixing Shen, Yue Lu, Jingyan Zheng, Peifen Fu

**Affiliations:** Department of Breast Surgery, The First Affiliated Hospital, Zhejiang University School of Medicine, Hangzhou, 310003, China; Department of Breast and Thyroid Surgery, Taizhou Hospital, Zhejiang University, Taizhou, Zhejiang, 318000, China; Department of Breast and Thyroid Surgery, Cixi People’s Hospital, Cixi, Zhejiang, 315300, China; Department of Breast and Thyroid Surgery, First Affiliated Hospital of Huzhou University, Huzhou, 313000, Zhejiang, China; Department of Breast and Thyroid Surgery, Lishui People’s Hospital, The Six Affiliated Hospital of Wenzhou Medical University, Lishui, Zhejiang, 323000, China

**Keywords:** EIF5A2, cancer, prognosis, meta-analysis, EMT

## Abstract

**Aims:**

In cancer biology, the aberrant overexpression of eukaryotic translation initiation factor 5A2 (EIF5A2) has been correlative with an ominous prognosis, thereby underscoring its pivotal role in fostering metastatic progression. Consequently, EIF5A2 has garnered significant attention as a compelling prognostic biomarker for various malignancies. Our research endeavors were thus aimed at elucidating the utility and significance of EIF5A2 as a robust indicator of cancer outcome prediction.

**Method:**

An exhaustive search of the PubMed, EMBASE, and Web of Science databases found relevant studies. The link between EIF5A2 and survival prognosis was examined using hazard ratios and 95% confidence intervals. Subsequently, The Cancer Genome Atlas (TCGA) and the Gene Expression Profiling Interactive Analysis (GEPIA) databases were employed to validate EIF5A2 expression across various cancer types.

**Results:**

Through pooled analysis, we found that increased EIF5A2 expression was significantly associated with decreased overall survival (OS) and disease-free survival/progression-free survival/relapse-free survival (DFS/PFS/RFS). Moreover, TCGA analysis revealed that EIF5A2 was significantly upregulated in 27 types of cancer, with overexpression being linked to shorter OS in three, worse DFS in two, and worse PFS in six types of cancer. GEPIA showed that patients with EIF5A2 overexpression had reduced OS and DFS.

**Conclusions:**

In solid tumors, EIF5A2 emerges as a reliable prognostic marker. Our meta-analysis comprehensively analyzed the prognostic value of EIF5A2 in solid tumors and assessed its efficacy as a predictive marker.

## 1 Introduction

Eukaryotic translation initiation factor 5A (EIF5A), an essential protein, plays a vital role in maintaining cellular polyamine homeostasis and influencing ribosomal peptidyl-transferase [1]. Eukaryotic translation initiation factor 5A2 (EIF5A2), a variant of EIF5A, enhances signal transducer and activator of transcription 3 (STAT3) entry into the nucleus. This, in turn, increases STAT3 enrichment on the promoter of transforming growth factor-β1 (TGF-β1), resulting in upregulated TGF-β1 expression and facilitating the epithelial–mesenchymal transition (EMT) [2]. EMT enables tumor cells to transition between epithelial and mesenchymal states, which is critical for cancer metastasis [3]. Elevated *EIF5A2* levels have been detected in various cancers, where it promotes cancer spread and presents as a promising target for cancer treatment [4]. Inhibition of *EIF5A2* has been shown to suppress tumor development and metastasis, while also overcoming chemotherapy resistance [5].

Previous studies suggest that high levels of EIF5A2 are associated with poor prognosis [6]. *EIF5A2* was identified in a primary ovarian cancer cell line, and its overexpression in ovarian tumor predicts poor prognosis [7]. Similar outcomes were observed in patients with bladder urothelial cancer (BUC) [8,9], upper tract urothelial carcinoma (UTUC) [10,11], and prostate cancer [12]. In gastrointestinal tumors such as oral squamous cell carcinoma (OSCC) [13], esophageal squamous cell carcinoma (ESCC) [14], gastric cancer (GC) [15,16], hepatocellular carcinoma (HCC) [17,18], gallbladder cancer (GBC) [19], intrahepatic cholangiocarcinoma (ICC) [2], pancreatic adenocarcinoma [20], and colorectal carcinoma (CRC) [21], overexpression of EIF5A2 is also a predictor of poor prognosis. Additionally, overexpression of EIF5A2 is associated with poor prognosis in melanoma [22], nasopharyngeal carcinoma (NPC) [23], cervical cancer [24], and non-small-cell lung cancer (NSCLC) [25] patients.

As scientific knowledge continues to expand, we gain a better understanding of the complex mechanisms underlying cancer metastasis. However, some aspects of these mechanisms remain elusive, underscoring the urgent need for continued research aimed at elucidating them and identifying novel biomarkers for cancer treatment. EIF5A2 is a promising candidate as a prognostic marker for cancer, but the findings from previous studies have not been consistent, making it difficult to establish its predictive significance definitively. To address this issue, in this study, we conducted a meta-analysis to evaluate the predictive value of EIF5A2 in solid tumors and assess its potential as a reliable predictive marker.

## 2 Materials & methods

### 2.1 Search strategy

We conducted a systematic search of the PubMed, Web of Science, and EMBASE databases to retrieve relevant publications up until February 10, 2023. The search utilized the keywords “EIF5A2” and “cancer” OR “carcinoma” OR “neoplasm” OR “tumor” OR “tumour,” along with “prognosis” OR “prognostic” OR “survival” OR “outcome.” No language restrictions were applied to the search. We reviewed titles, abstracts, full-text manuscripts, and references to identify relevant studies. As this study did not involve human participants, informed consent was not required (Table 1).

**Table 1:** Search strings

Database	Search string	Number of studies
Web of Science	(“EIF5A2”) and (“cancer” OR “carcinoma” OR “neoplasm” OR “tumor” OR “tumour”) and (“prognosis” OR “prognostic” OR “survival” OR “outcome”)	83
PubMed	(“EIF5A2”) and (“cancer” OR “carcinoma” OR “neoplasm” OR “tumor” OR “tumour”) and (“prognosis” OR “prognostic” OR “survival” OR “outcome”)	66
Embase	(“EIF5A2”) and (“cancer” OR “carcinoma” OR “neoplasm” OR “tumor” OR “tumour”) and (“prognosis” OR “prognostic” OR “survival” OR “outcome”)	75
Total		224

EIF5A2: eukaryotic translation initiation factor 5A2.

### 2.2 Study selection

We included publications that investigated the relationship between EIF5A2 and survival prognosis in solid tumors, reported measurements of EIF5A2 expression in tissue or blood, and provided sufficient data to calculate hazard ratios (HRs) and 95% confidence intervals (CIs). Our study encompasses a variety of detection methods, ensuring that a broad spectrum of research is included. While real-time PCR is acknowledged as a common method, other detection techniques were also considered, enhancing the diversity of the literature reviewed. Studies that did not provide enough information to estimate HRs and 95% CIs, as well as reviews, case reports, letters, abstracts, animal studies, public database datasets, and duplicated or overlapped research, were excluded from our analysis. Literature in languages other than English or Chinese was excluded due to limitations in linguistic proficiency. Our study prioritized articles that explicitly reported HRs in their findings. Articles relying solely on Kaplan–Meier curves for survival analysis were intentionally excluded from our study.

### 2.3 Data extraction & quality assessment

We extracted relevant data from each eligible study, including author name, publication year, and country of sample origin. Additionally, we collected information on the type of tumor samples, sample size, detection methods, and other characteristics. We also obtained overall survival (OS), disease-free survival/progression-free survival/relapse-free survival (DFS/PFS/RFS), HRs, and their corresponding 95% CIs. When available, multivariate analysis was preferred over univariate analysis for increased precision. The quality of each study was assessed using the Newcastle–Ottawa Quality Assessment Scale to evaluate its effectiveness.

### 2.4 Statistical analysis

We used HRs and their corresponding 95% CIs to calculate the pooled data in our analysis, directly utilizing the values reported in each study. To assess heterogeneity, *I* ^2^ or *p*-value was used. When *I* ^2^ was less than 50% or *p*-value was larger than 0.05, a fixed-effects model was employed, and when *I* ^2^ was greater than 50% or *p*-value was less than 0.05, a random-effects model was employed. Sensitivity analysis was conducted to assess the reliability of the results. To assess publication bias, funnel plots and Egger’s test were used. STATA 17.0 software was used for all data analyses (Stata Corporation, TX, USA). *P*-values lower than 0.05 were deemed statistically significant.

### 2.5 Bioinformatics analysis

We collected RNA-sequencing expression (level 3) profiles and corresponding clinical information from The Cancer Genome Atlas (TCGA) database (https://portal.gdc.cancer.gov/) for 10,030 patients with various types of cancer. Additionally, we obtained mRNA expression data from paired normal tissue samples in these tumors. Normal tissue samples were also retrieved from the GTEx V8 release version (https://gtexportal.org/home/datasets) for comparison. We used univariate Cox regression analysis and the “forestplot” R package in R version 4.0.3 to display *p*-values, HRs, and their respective 95% CIs for each variable. The Gene Expression Profiling Interactive Analysis (GEPIA) tool (http://gepia.cancer-pku.cn/), based on TCGA and GTEx data, was used to evaluate abnormal EIF5A2 expression in cancer tissues. We then obtained survival plots in the form of Kaplan–Meier curves for the association between EIF5A2 expression and OS or DFS. *P*-values less than 0.05 were considered statistically significant.

## 3 Results

### 3.1 Search results

**Figure 1:**
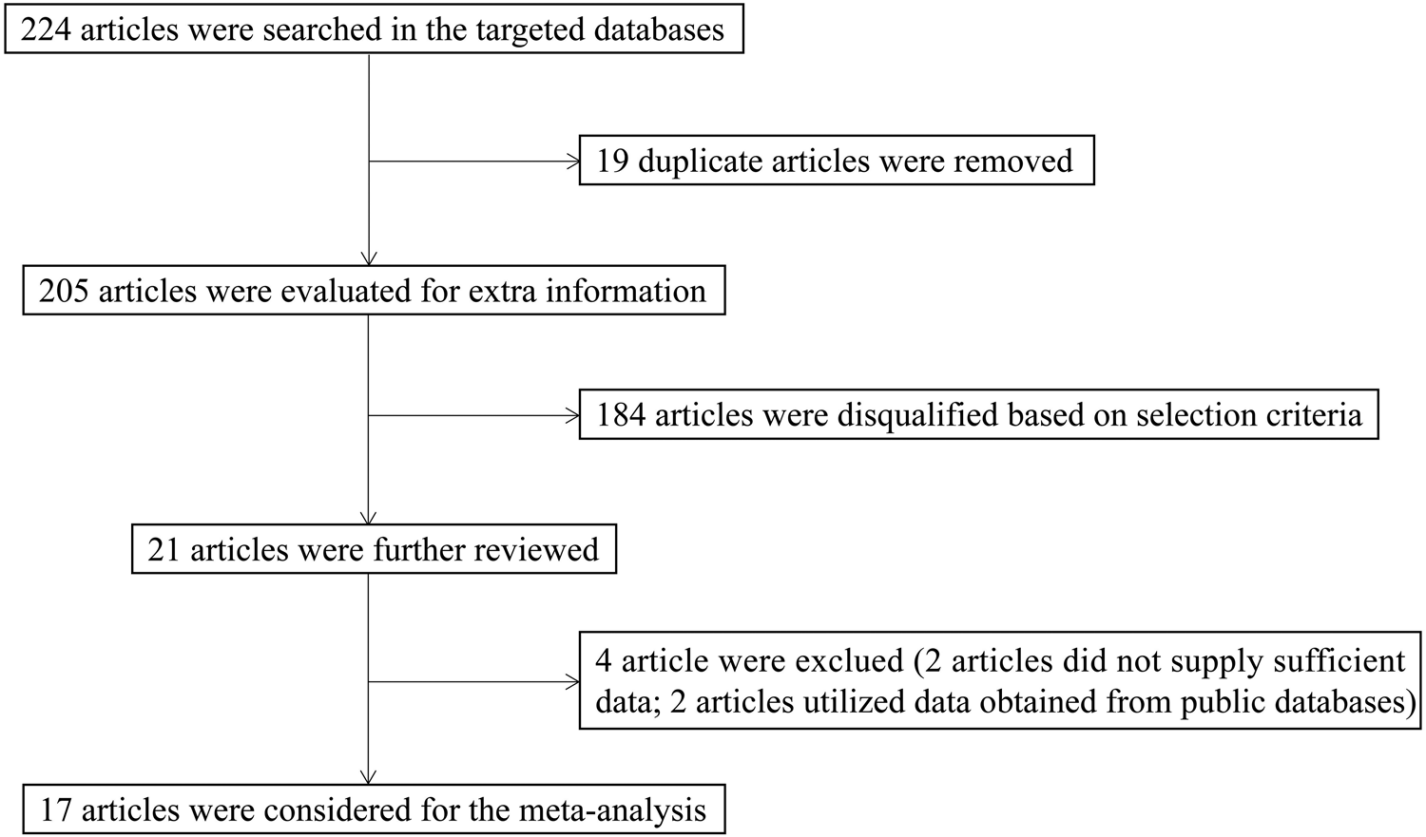
Literature search process flowchart.

Our search strategy yielded a total of 224 articles from the designated databases. After removing 19 duplicates, we screened 205 articles for additional information using our selection criteria, resulting in the elimination of 184 articles and leaving 21 articles for further screening. Of these, two articles did not provide sufficient data, and two others used data obtained from public databases. Ultimately, our meta-analysis included 17 articles published between 2009 and 2022. Figure 1 illustrates the search strategy flowchart.

### 3.2 Study characteristics

A total of 3,554 samples were included in our meta-analysis, with sample sizes ranging from 72 to 436 per study. Various malignancies were investigated, including ovarian cancer, BUC, UTUC, melanoma, prostate cancer, OSCC, ESCC, GC, HCC, GBC, ICC, pancreatic adenocarcinoma, CRC, NPC, cervical cancer, and NSCLC. In 20 studies, the overexpression of EIF5A2 was detected in tissue samples using immunohistochemistry. Table 2 provides fundamental information regarding the included literature.

**Table 2:** Elements of relevant studies

Study	Year	Country	Race	Sample size	Tumor type	Detected method	Detected sample	Survival analysis	NOS score
Chen et al. [8]	2009	China	Asian	86	Bladder urothelial carcinoma	IHC	Tissue	OS	7
Fang et al. [11]	2019	China	Asian	101	Upper urinary tract urothelial carcinoma	IHC	Tissue	OS, PFS	7
He et al. [25]	2011	China	Asian	224	Non-small-cell lung cancer	IHC	Tissue	OS	6
Huang et al. [10]	2018	China	Asian	109	Upper urinary tract urothelial carcinoma	IHC	Tissue	RFS	7
Huang et al. [23]	2016	China	Asian	123	NPC	IHC	Tissue	OS	7
Khosravi et al. [22]	2016	Canada	Caucasian	382	Melanoma	IHC	Tissue	OS	6
Lin et al. [13]	2020	China	Asian	272	OSCC	IHC	Tissue	OS	6
Luo et al. [9]	2009	China	Asian	112	Bladder urothelial carcinoma	IHC	Tissue	PFS, RFS	6
Meng et al. [16]	2015	China	Asian	160/145	GC	IHC	Tissue	OS, DFS	5
Wang et al. [17]	2014	China	Asian	212	Hepatocellular carcinoma	IHC	Tissue	OS	7
Wei et al. [20]	2013	China	Asian	73	Pancreatic adenocarcinoma	IHC	Tissue	OS	5
Wei et al. [26]	2014	China	Asian	154	Bladder cancer	IHC	Tissue	OS	6
Yang et al. [7]	2009	China	Asian	110	Ovarian tumor	IHC	Tissue	OS	6
Yang et al. [15]	2016A	China	Asian	436	GC	IHC	Tissue	OS	6
Yang et al. [24]	2016B	China	Asian	314	Cervical cancer	IHC	Tissue	OS, DFS	7
Zheng et al. [19]	2020	China	Asian	80	GBC	IHC	Tissue	OS	7
Zhu et al. [21]	2011	China	Asian	229	CRC	IHC	Tissue	OS	7

IHC: immunohistochemistry; OS: overall survival; DFS: disease-free survival; PFS: progression-free survival; RFS: relapse-free survival.

### 3.3 High EIF5A2 expression & OS

**Figure 2:**
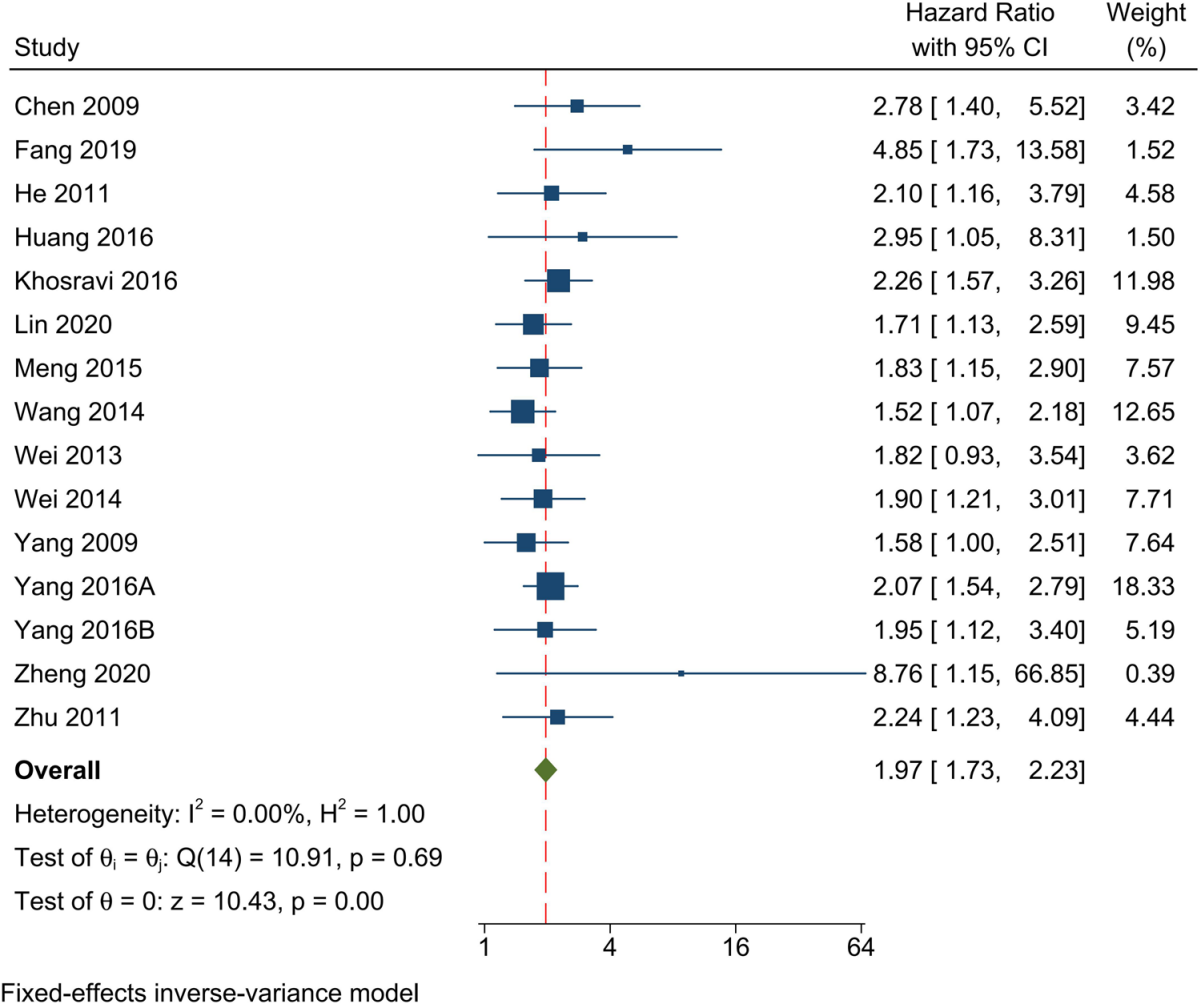
Forest plot of the relationship between overexpression of EIF5A2 and OS.

Fourteen studies examined the association between high EIF5A2 expression and prognosis using OS. As there was no significant heterogeneity observed in this analysis (*I* ^2^ = 0), a fixed-effects model was used to estimate the pooled HR with a 95% CI. The results showed that high EIF5A2 expression was substantially linked with shorter OS (HR: 1.97; 95% CI: 1.73–2.22), as depicted in Figure 2.

### 3.4 Subgroup analysis for OS

Subgroup analyses were conducted according to tumor type, race, and sample size. The findings of these subgroup analyses are presented in Table 3. In terms of tumor types, high EIF5A2 expression was associated with poor OS in digestive system tumors, with an HR of 1.89 (95% CI: 1.57–2.27). Similarly, gynecological and reproductive system tumors had an HR of 1.72 (95% CI: 2.21–2.45). Head and neck cancers exhibited an HR of 1.85 (95% CI: 1.26–2.71), and melanoma had an HR of 2.26 (95% CI: 1.57–3.26). Respiratory system tumors had an HR of 2.10 (95% CI: 1.16–3.79), and urinary system tumors had an HR of 2.52 (95% CI: 1.58–4.03). When considering race, both Asian and Caucasian populations showed statistically significant HRs. Asian individuals had an HR of 1.93 (95% CI: 1.68–2.21), while Caucasians had an HR of 2.26 (95% CI: 1.57–3.26). Finally, the analysis of sample sizes revealed that studies with less than 200 participants had an HR of 2.16 (95% CI: 1.62–2.89), while those with more than 200 participants had an HR of 1.92 (95% CI: 1.66–2.23).

**Table 3:** Subgroup analysis for OS

Clinical features	Studies (*n*)	Pooled HR (95% CI)	*p*-value	Heterogeneity		
				*I* ^2^ (%)	*p*-value	Model
**Tumor type**						
Digestive system	6	1.89 (1.57–2.27)	<0.001	0	0.506	Random
Gynecological and Reproductive systems	2	1.72 (2.21–2.45)	0.003	0	0.573	Random
Head and neck cancers	2	1.85 (1.26–2.71)	0.002	0	0.340	Random
Melanoma	1	2.26 (1.57–3.26)	<0.001			
Respiratory system	1	2.10 (1.16–3.79)	0.014			
Urinary system	3	2.52 (1.58–4.03)	<0.001	31.9	0.230	Random
**Race**						
Asian	14	1.93 (1.68–2.21)	<0.001	0	0.671	Random
Caucasian	1	2.26 (1.57–3.26)	<0.001			
**Sample size**						
<200	7	2.16 (1.62–2.89)	<0.001	17.7	0.295	Random
≥200	8	1.92 (1.66–2.23)	<0.001	0	0.855	Random

HR: Hazard ratio.

### 3.5 High EIF5A2 expression & DFS/PFS/RFS

**Figure 3:**
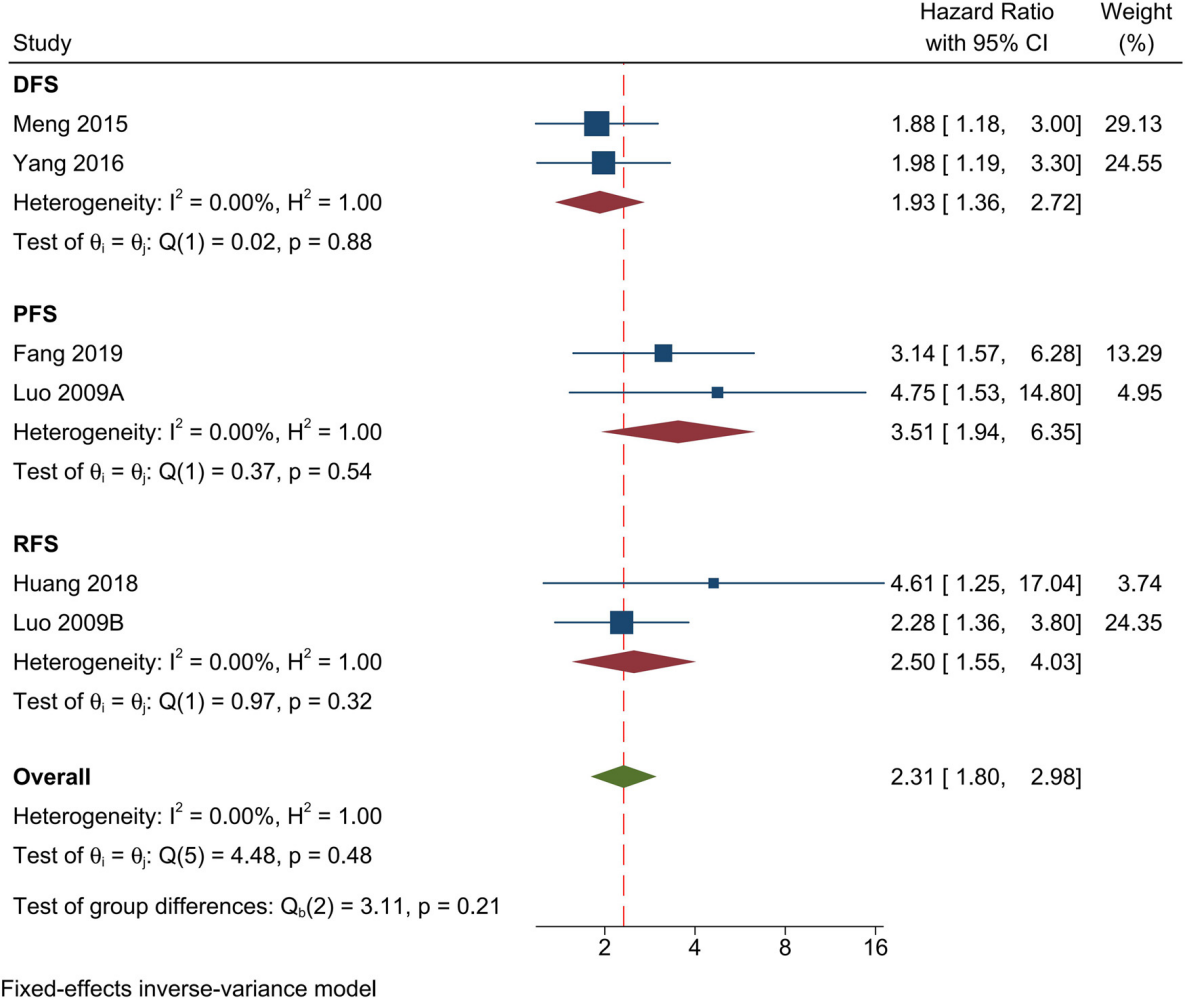
Forest plot of the relationship between overexpression of EIF5A2 and DFS/PFS/RFS.

Six studies examined the connection between overexpression of EIF5A2 and prognosis using DFS/PFS/RFS. Using the fixed-effects model (*I* ^2^ = 0), a comprehensive analysis revealed that increased EIF5A2 expression was substantially related to decreased DFS/PFS/RFS (HR: 2.31; 95% CI: 1.80–2.98) (Figure 3). Additionally, we independently analyzed DFS, PFS and RFS results. High EIF5A2 expression was connected with decreased DFS (HR: 1.93; 95% CI: 1.36–2.72), PFS (HR: 3.51; 95% CI: 1.94–6.35), and RFS (HR: 2.50; 95% CI: 1.55–4.03).

### 3.6 High EIF5A2 expression & lymph node metastasis

**Figure 4:**
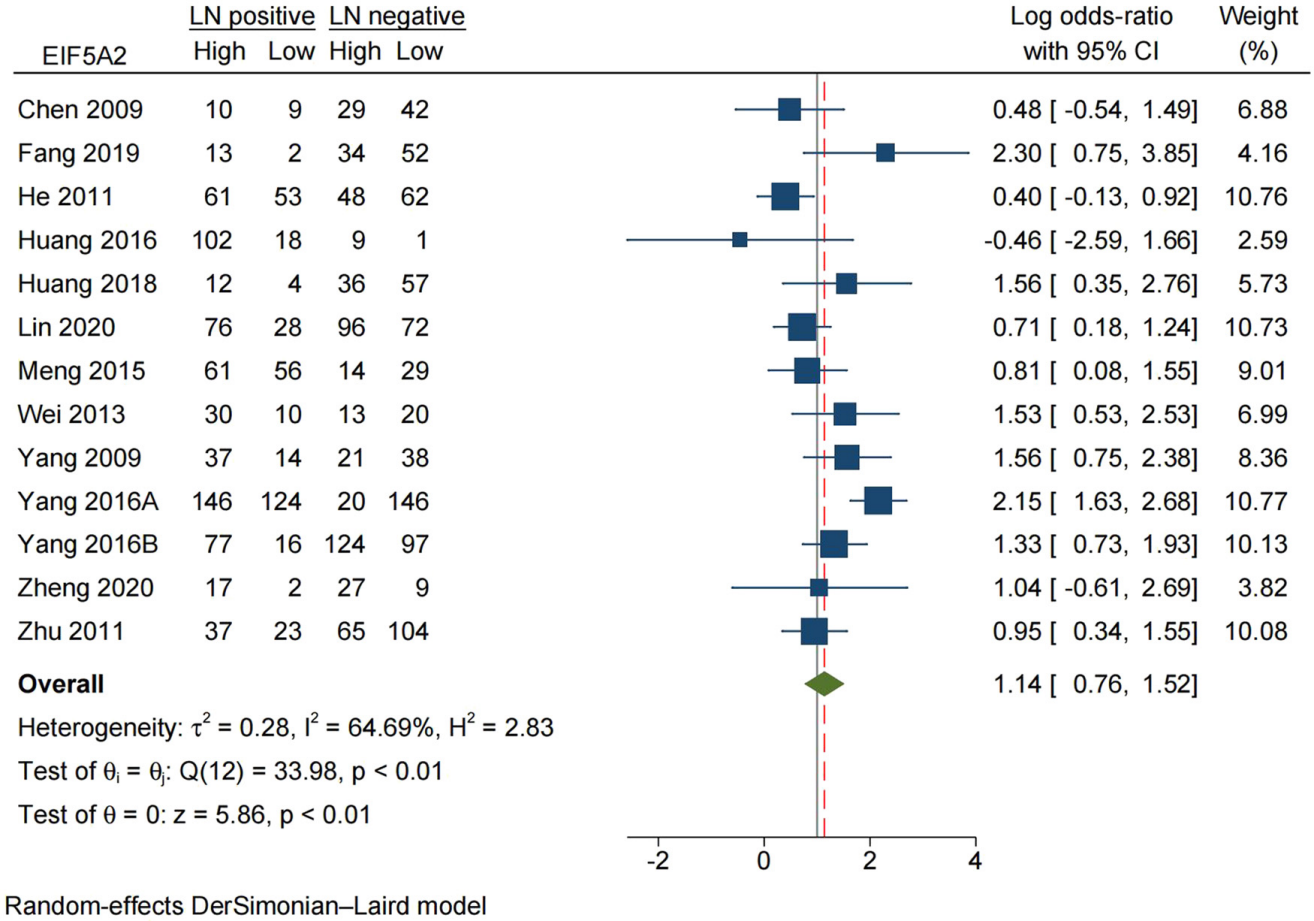
Forest plot of the relationship between expression of EIF5A2 and lymph node metastasis: LN, lymph node.

To further explore the association between EIF5A2 and lymph node metastasis, we performed a thorough analysis by compiling data on high EIF5A2 expression and lymph node (LN) metastasis status. The findings indicated there is no association between elevated EIF5A2 expression and LN status (LN positive vs LN negative), as evidenced by an odds ratio (OR) of 1.14 (95% CI: 0.76–1.52) (Figure 4).

### 3.7 Sensitivity analysis

**Figure 5:**
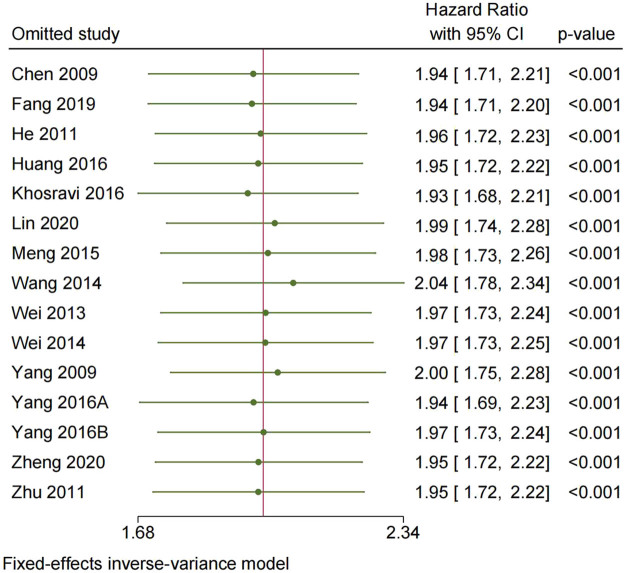
Sensitivity analysis for OS.

**Figure 6:**
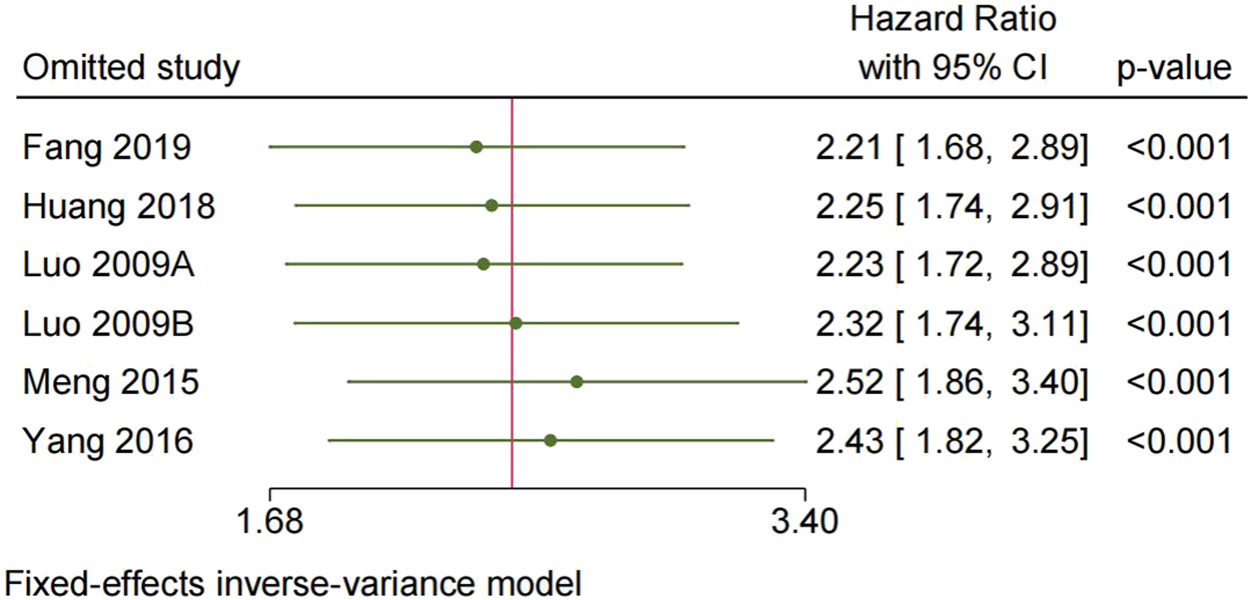
Sensitivity analysis for DFS/PFS/RFS.

To assess the robustness of the findings, a sensitivity analysis was conducted by removing each study individually. The results, as depicted in Figures 5 and 6, did not show any significant alteration from the overall analysis, indicating the stability of the results.

### 3.8 Publication bias

**Figure 7:**
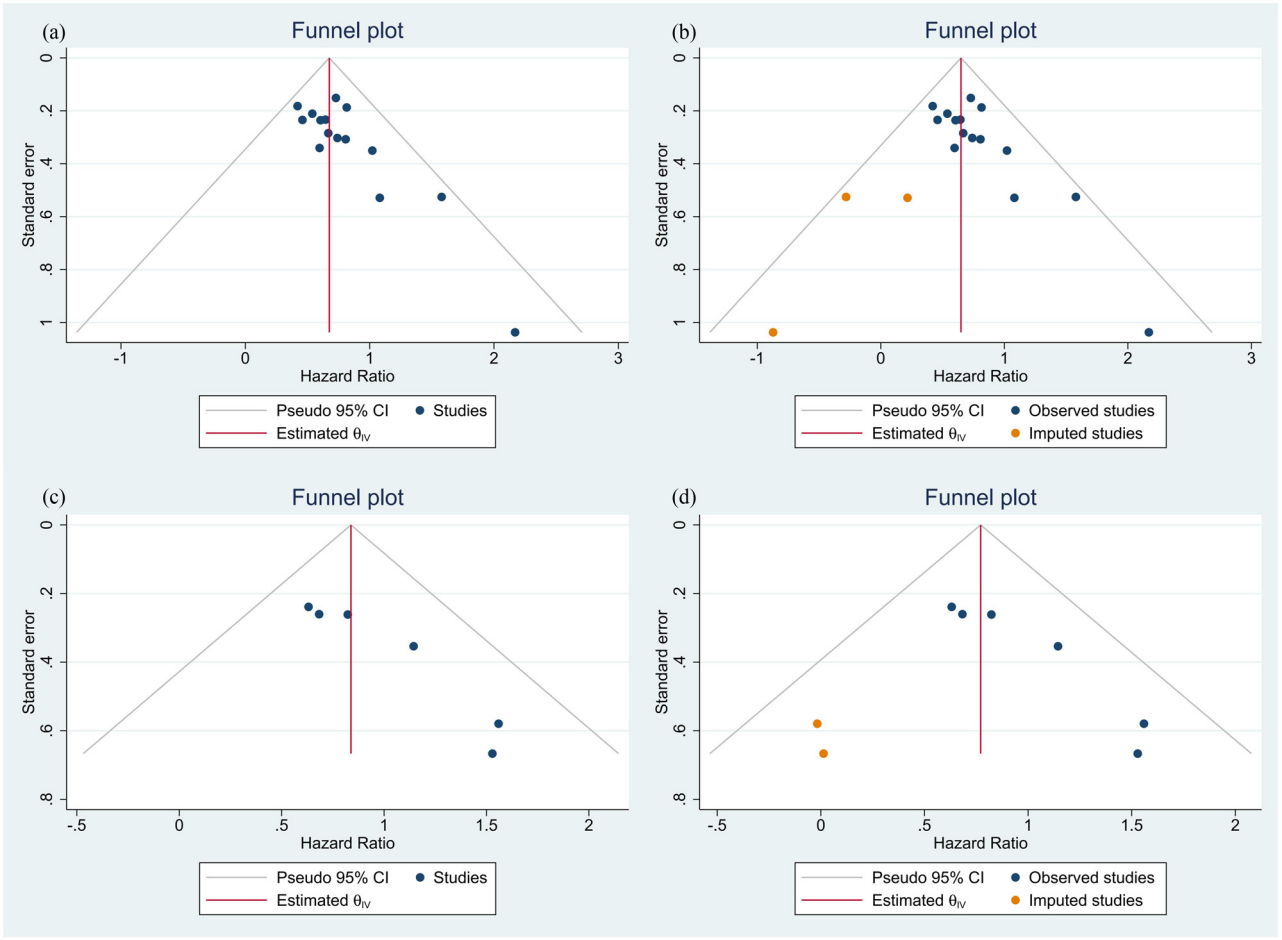
Funnel plots for publication bias: (a) funnel plots for OS; (b) filled funnel plot for OS; (c) funnel plots for DFS/PFS/RFS; (d) filled funnel plot for DFS/PFS/RFS.

**Figure 8:**
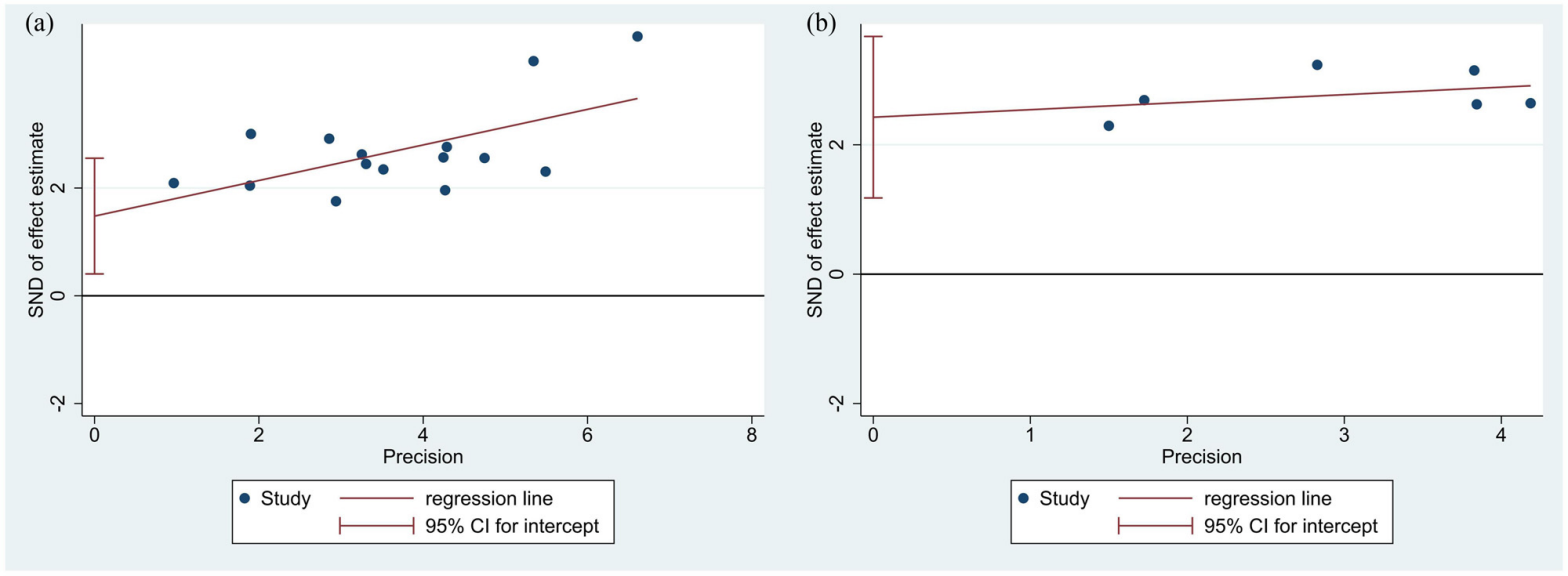
(a) Egger’s test for OS data, and (b) Egger’s test for DFS/PFS/RFS data.

To assess publication bias for OS or DFS/PFS/RFS, the study used funnel plots and Egger’s test to generate statistical evidence (Figure 7). The results indicated a significant publication bias, with Egger’s test *p*-values of 0.011 for OS (Figure 8a) and 0.006 for DFS/PFS/RFS (Figure 8b). To further examine publication bias, the study employed the trim-and-fill strategy. It was found that the pooled HRs for OS and DFS/PFS/RFS were 1.914 (95% CI: 1.689–2.168) and 2.162 (95% CI: 1.697–2.755), respectively, which demonstrated that the meta-analysis results remained robust despite the presence of publication bias.

### 3.9 Verification in bioinformatics databases

**Figure 9:**
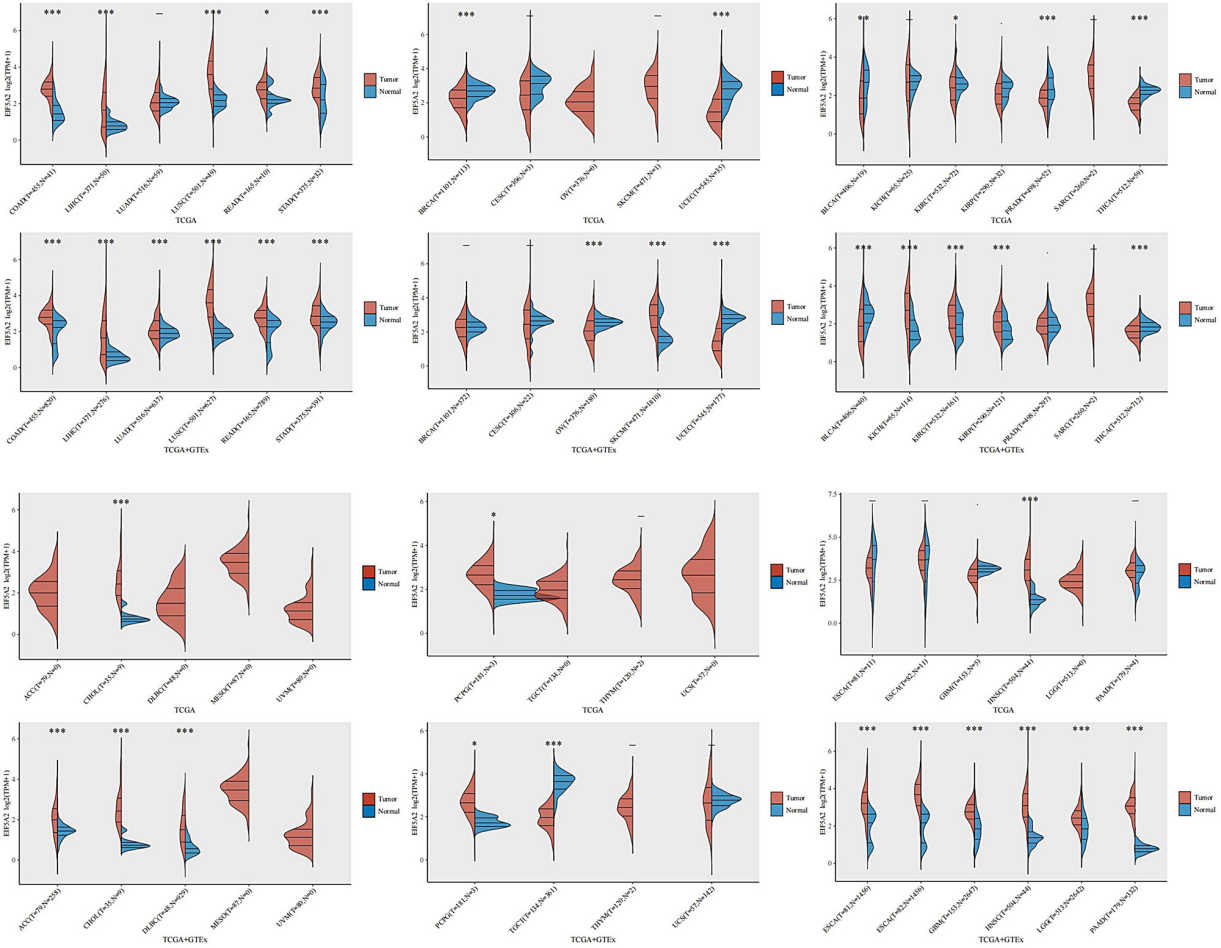
EIF5A2 expression in different types of cancers. The expression distribution of EIF5A2 in tumor tissues and normal tissues. The abscissa represents different tumor tissues, the ordinate represents the expression distribution of EIF5A2, and different colors represent different groups. *p<  0.05, **p<  0.01,***p<  0.001, asterisks (*) stand for significance levels. The statistical difference of two groups was compared through the Wilcox test. ACC: adrenocortical carcinoma; BLCA: bladder urothelial carcinoma; BRCA: breast invasive carcinoma; CESC: cervical squamous cell carcinoma and endocervical adenocarcinoma; CHOL: cholangiocarcinoma; COAD: colon adenocarcinoma; ESCA: esophageal carcinoma; GBM: glioblastoma multiforme; HNSC: head and neck squamous cell carcinoma; KICH: kidney chromophobe; KIRC: kidney renal clear cell carcinoma; KIRP: kidney renal papillary cell carcinoma; LGG: brain lower-grade glioma; LIHC: liver hepatocellular carcinoma; LUAD: lung adenocarcinoma; LUSC: lung squamous cell carcinoma; MESO: mesothelioma; OV: ovarian serous cystadenocarcinoma; PAAD: pancreatic adenocarcinoma; PCPG: pheochromocytoma and paraganglioma; PRAD: prostate adenocarcinoma; READ: rectum adenocarcinoma; SARC: sarcoma; SKCM: skin cutaneous melanoma; STAD: stomach adenocarcinoma; TGCT: testicular germ cell tumors; THCA: thyroid carcinoma; THYM: thymoma; UCEC: uterine corpus endometrial carcinoma; UCS: uterine carcinosarcoma; UVM: uveal melanoma.

**Figure 10:**
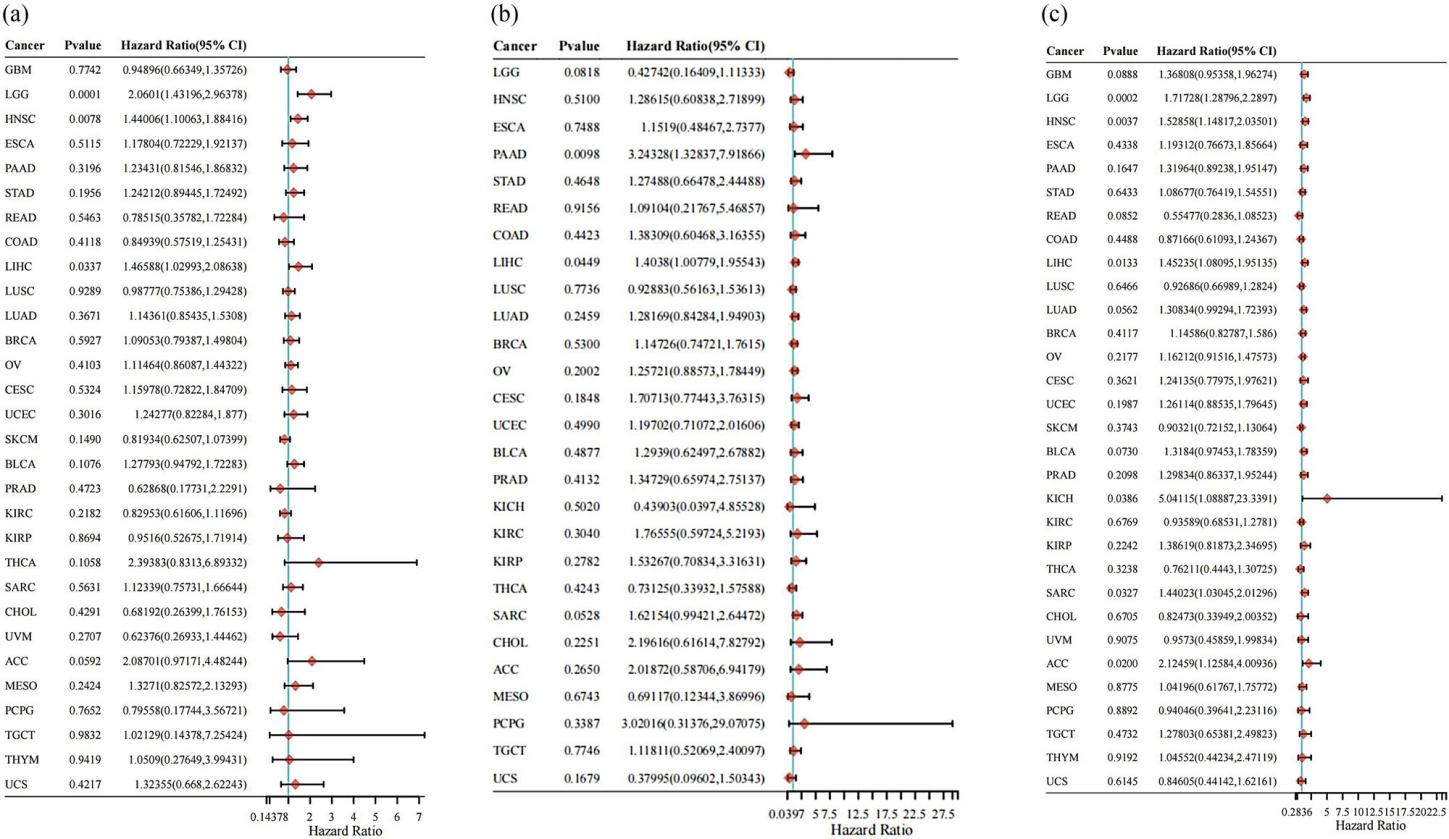
Forest plot for OS (a), DFS (b), and PFS (c). The *p*-value, risk coefficient (HR), and CI of EIF5A2 in multiple tumors are analyzed by univariate cox regression. ACC: adrenocortical carcinoma; BLCA: bladder urothelial carcinoma; BRCA: breast invasive carcinoma; CESC: cervical squamous cell carcinoma and endocervical adenocarcinoma; CHOL: cholangiocarcinoma; COAD: colon adenocarcinoma; ESCA: esophageal carcinoma; GBM: glioblastoma multiforme; HNSC: head and neck squamous cell carcinoma; KICH: kidney chromophobe; KIRC: kidney renal clear cell carcinoma; KIRP: kidney renal papillary cell carcinoma; LGG: brain lower-grade glioma; LIHC: liver hepatocellular carcinoma; LUAD: lung adenocarcinoma; LUSC: lung squamous cell carcinoma; MESO: mesothelioma; OV: ovarian serous cystadenocarcinoma; PAAD: pancreatic adenocarcinoma; PCPG: pheochromocytoma and paraganglioma; PRAD: prostate adenocarcinoma; READ: rectum adenocarcinoma; SARC: sarcoma; SKCM: skin cutaneous melanoma; STAD: stomach adenocarcinoma; TGCT: testicular germ cell tumors; THCA: thyroid carcinoma; THYM: thymoma; UCEC: uterine corpus endometrial carcinoma; UCS: uterine carcinosarcoma; UVM: uveal melanoma.

**Figure 11:**
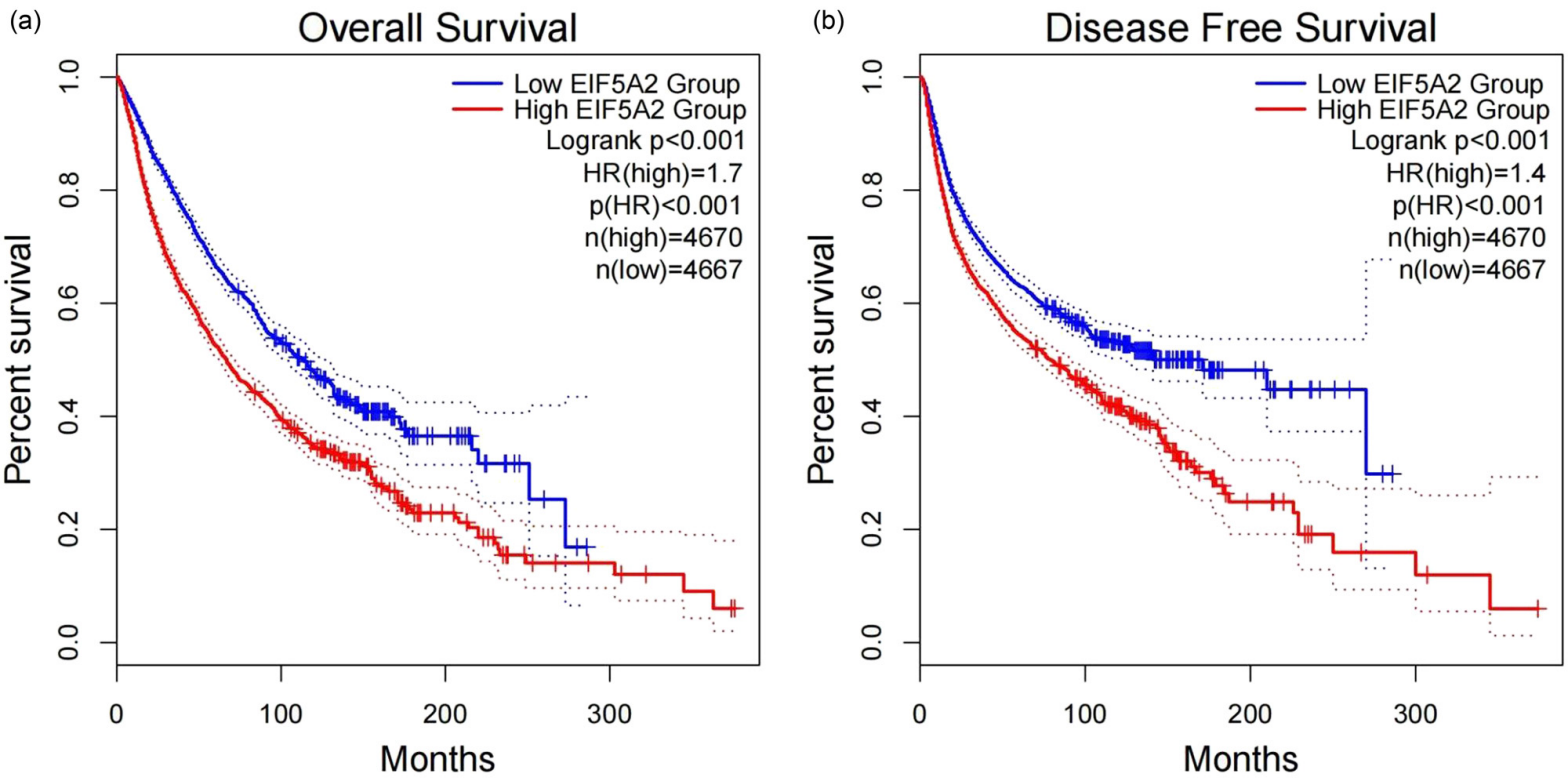
Kaplan–Meier curves for OS (a) and DFS (b). The prognostic value of EIF5A2 for the OS and DFS of patients with cancer using the GEPIA database. HR: hazard ratio; OS: overall survival; DFS: disease-free survival.

To further confirm our findings, we investigated if EIF5A2 could serve as a prognostic biomarker across various types of cancer. Our results demonstrated that the expression of EIF5A2 was significantly different in 27 types of cancers (*p* < 0.05; Figure 9) compared to healthy tissues. Additionally, univariate Cox regression analyses were conducted to evaluate the prognostic value of EIF5A2 in a wide range of malignancies. The results showed that the overexpression of EIF5A2 was associated with poor OS in three types of cancer (p < 0.05; Figure 10a) and worse DFS and PFS in two and six cancer types (p < 0.05; Figure 10b and c). Furthermore, we used the GEPIA online tool to assess EIF5A2 expression across 31 types of cancers. The patients were divided into EIF5A2 high and low expression groups based on the median value, and the results (Figure 11) confirmed that EIF5A2 overexpression was linked to shorter OS and DFS in patients with cancer. These findings, which were consistent with the conclusions of our meta-analysis, suggest that EIF5A2 could be a promising prognostic biomarker for various types of cancer.

## 4 Discussion

Metastasis, the most deleterious hallmark of cancer, remains responsible for a substantial proportion of cancer-related deaths [27]. The interplay between autophagy and EMT, two critical processes that govern cellular behavior, has emerged as an underlying molecular mechanism driving tumorigenesis and metastasis [28]. Notably, androgen receptor (AR) signaling governs the expression of EIF5A2 in androgen-dependent cells, promoting prostate cancer metastasis by inducing EMT and elevating EIF5A2 expression [29]. Autophagy, an adaptive stress response that degrades unwanted organelles and biomolecules, contributes to the immunosuppressive environment that facilitates tumor initiation and progression [30].

Drug resistance in cancer cells reduces the effectiveness of current treatments for many types of malignancy, including chemotherapy and targeted therapies [31]. Many studies have studied the function of EMT in tumor drug resistance, and various EMT-mediated signaling pathways are involved in drug resistance [32]. EIF5A2 has been implicated in promoting drug resistance in various malignancies. In HCC, for instance, elevated EIF5A2 levels mediate chemo-resistance by suppressing autophagy-mediated cell death [18]. Hypoxia further amplifies EIF5A2 expression in NSCLC, thereby promoting cisplatin resistance via autophagy induction [33]. Similarly, in breast cancer cells, overexpression of EIF5A2 correlates with lower sensitivity to doxorubicin [34]. In addition, recent studies suggest that EIF5A2 might regulate cellular aging by modulating transcriptional activity, adding another layer of complexity to its diverse roles in various biological processes [35].

Given the critical involvement of EIF5A2 in tumor-specific mechanisms such as EMT, autophagy, and drug resistance, it represents a promising target for developing novel therapeutic approaches. A meta-analysis conducted in our study corroborates the association between EIF5A2 overexpression and poor prognosis in solid tumors, underscoring the potential of EIF5A2 as a reliable and informative biomarker of malignancy outcome.

This meta-analysis is subject to several limitations. First, the sample sizes of all included studies were relatively small, and hence, the accuracy of their data may be compromised. Second, clinical characteristics of the studies were not made available. Third, there exists a notable publication bias for survival outcomes, potentially resulting from variations in research methodologies, clinical experience of authors, statistical analysis, and adjustment factors. Finally, most of the retrospective investigations were conducted in Asia, thereby limiting the generalizability of the outcomes to other regions.

As previously discussed, EIF5A2 has been implicated in tumor initiation, progression, metastasis, and chemotherapy resistance, making it a promising prognostic marker for solid malignancies. Robust prognostic markers not only enable personalized treatment for each patient by allowing for the early identification of high- and low-risk individuals, but also improve overall clinical outcomes. Despite the potential significance of EIF5A2 as a prognostic biomarker, its clinical relevance in solid tumors is still not well established. Thus, our meta-analysis aimed to comprehensively explore the potential clinical utility of EIF5A2 in solid malignancies.
